# Biopsies of osseous jaw lesions using 3D-printed surgical guides: a clinical study

**DOI:** 10.1186/s40001-022-00726-8

**Published:** 2022-07-02

**Authors:** Lukas Postl, Thomas Mücke, Stefan Hunger, Sabina Noreen Wuersching, Svenia Holberg, Oliver Bissinger, Rainer Burgkart, Michael Malek, Stefan Krennmair

**Affiliations:** 1 Medical Faculty, Johannes Kepler University Linz, Altenberger Strasse 69, 4040 Linz, Austria; 2 NumBioLab, Ludwig-Maximilians University of Munich, Munich, Germany; 3 Department of Oral and Maxillo-Facial Surgery, Klinikum Rechts Der Isar, Technische Universitaet Muenchen, Munich, Germany; 4 Department of Conservative Dentistry and Periodontology, University Hospital, LMU Munich, Munich, Germany; 5 Department of Oral and Maxillofacial Surgery, Medizinische Universitaet Innsbruck, Innsbruck, Austria; 6 Clinic of Orthopaedics and Sportorthopaedics, Klinikum Rechts Der Isar, Technische Universitaet Muenchen, Munich, Germany; 7 Clinic of Oral and Maxillofacial Surgery, Kepler University Hospital, Johannes Kepler University, Linz, Austria

**Keywords:** 3D-printed surgical guide, Computer-assisted surgery, Computer-guided surgery, Stereolithography, Biopsy, 3D-printed bone models

## Abstract

**Background:**

Bone biopsies are often necessary to make a diagnosis in the case of irregular bone structures of the jaw. A 3D-printed surgical guide may be a helpful tool for enhancing the accuracy of the biopsy and for ensuring that the tissue of interest is precisely removed for examination. This study was conducted to compare the accuracy of biopsies performed with 3D-printed surgical guides to that of free-handed biopsies.

**Methods:**

Computed tomography scans were performed on patients with bony lesions of the lower jaw. Surgical guides were planned via computer-aided design and manufactured by a 3D-printer. Biopsies were performed with the surgical guides. Bone models of the lower jaw with geometries identical to the patients’ lower jaws were produced using a 3D-printer. The jaw models were fitted into a phantom head model and free-handed biopsies were taken as controls. The accuracy of the biopsies was evaluated by comparing the parameters for the axis, angle and depth of the biopsies to the planned parameters.

**Results:**

Eight patients were included. The mean deviation between the biopsy axes was significantly lower in guided procedures than in free-handed biopsies (1.4 mm ± 0.9 mm; 3.6 mm ± 1.0 mm; *p* = 0.0005). The mean biopsy angle deviation was also significantly lower in guided biopsies than in free-handed biopsies (6.8° ± 4.0; 15.4° ± 3.6; *p* = 0.0005). The biopsy depth showed no significant difference between the guided and the free-handed biopsies.

**Conclusions:**

Computer-guided biopsies allow significantly higher accuracy than free-handed procedures.

## Background

Aside from diagnostic imaging and clinical laboratory tests [1], biopsies are essential diagnostic measures for pathologies [2, 3]. Clinicians may face many challenges when performing osseous biopsies on the lower jaw due to its individual anatomy and the presence of anatomical structures such as nerves and tooth roots [4]. In order to avoid damage to these structures, the use of computer-assisted methods may be helpful [5]. Navigation systems are available for surgical procedures in the head area. However, most navigation systems require a registration process to locate the position of the anatomical structures of interest (in this case the lower jaw). Many navigation systems use markers to track the position of the anatomical structure as the patient moves. Since the mandible is mobile in the temporomandibular joint, it would make sense to attach markers in the bone of the mandible or on the teeth.

On the other hand, a surgical template may be useful, which is a common auxiliary tool used for implant positioning in dentistry and does not require such a registration process.

In dental implantology, it is still a widespread practice to produce the surgical guides using conventional methods in dental laboratories. The surface information of the teeth obtained from a plaster model usually suffices for determining or estimating the correct axis for implant placement [6]. In contrast, an osseous biopsy not only requires the surface information, but also the information about the location of the bony lesion, which in most cases is covered by soft tissues. For this purpose, the computer-aided design (CAD) technology is a helpful tool [7], enabling a fusion of these two data [8] which can be used to plan a precise surgical guide. CAD provides a complex 3D body of the surgical guide that can be best realized using computer-aided manufacturing (CAM) technology. CAD/CAM technology has been receiving extensive attention in the recent years [6, 9–11]. Nowadays, there are many systems on the market that allow a computer-assisted implementation using data obtained from CT scans [12–15]. While computer-assisted surgeries are gaining popularity among surgeons for implant positioning [16–18], they are not yet established as standard procedures for osseous biopsies of the jaw. Recently, a study demonstrated that templates for mandibular biopsies can be designed with implant planning software and the surgical guides were printed in collaboration with the medical industry [19, 20]. A retrospective analysis by Lotz et al. examined the angular deviation (angle between the planned biopsy channel and actual biopsy channel in degrees) and the deviation of the depth of the biopsies. However, no data on the deviation of the biopsy channels (distance between planned biopsy channel and actual biopsy channel in millimeters) were included [20].

In the recent years, a stereolithography 3D-printer (Form 2, Formlabs Inc., Somerville, Massachusetts, USA) was introduced allowing in-house printing of surgical resin templates, which saves time and resources. Among several studies examining the accuracy of computer-assisted systems in oral implantology, some in fact used surgical guides which were produced via stereolithographic 3D-printing [13, 21–23].

Therefore, it is of interest whether 3D-printed surgical guides are also suitable for performing osseous biopsies of the jaw. This, however, requires thorough studies on the accuracy of such computer-assisted biopsies. In a preliminary work, the precision of biopsies taken from lower jaw models using 3D-printed surgical templates was examined. The results showed that the usage of the 3D-printed surgical templates significantly increased the accuracy of the biopsies (deviation of axes and angular deviation) compared to biopsies performed free-handed in an experimental setting [5].

The present prospective study aims to evaluate the benefit of 3D-printed surgical templates for biopsies taken from patients’ lower jaws and compares the accuracy to free-handed biopsies. We hypothesize (null hypothesis) that there is no difference between the accuracy of computer-assisted biopsies and free-handed biopsies of the control group.

## Methods

### Study design and patient population

For this study, patients with an osseous lesion of the lower jaw and an indication for a biopsy were recruited. This study was approved by the local ethics committee (reference number D-41-17).

### Preoperative scans and planning

**Fig. 1:**
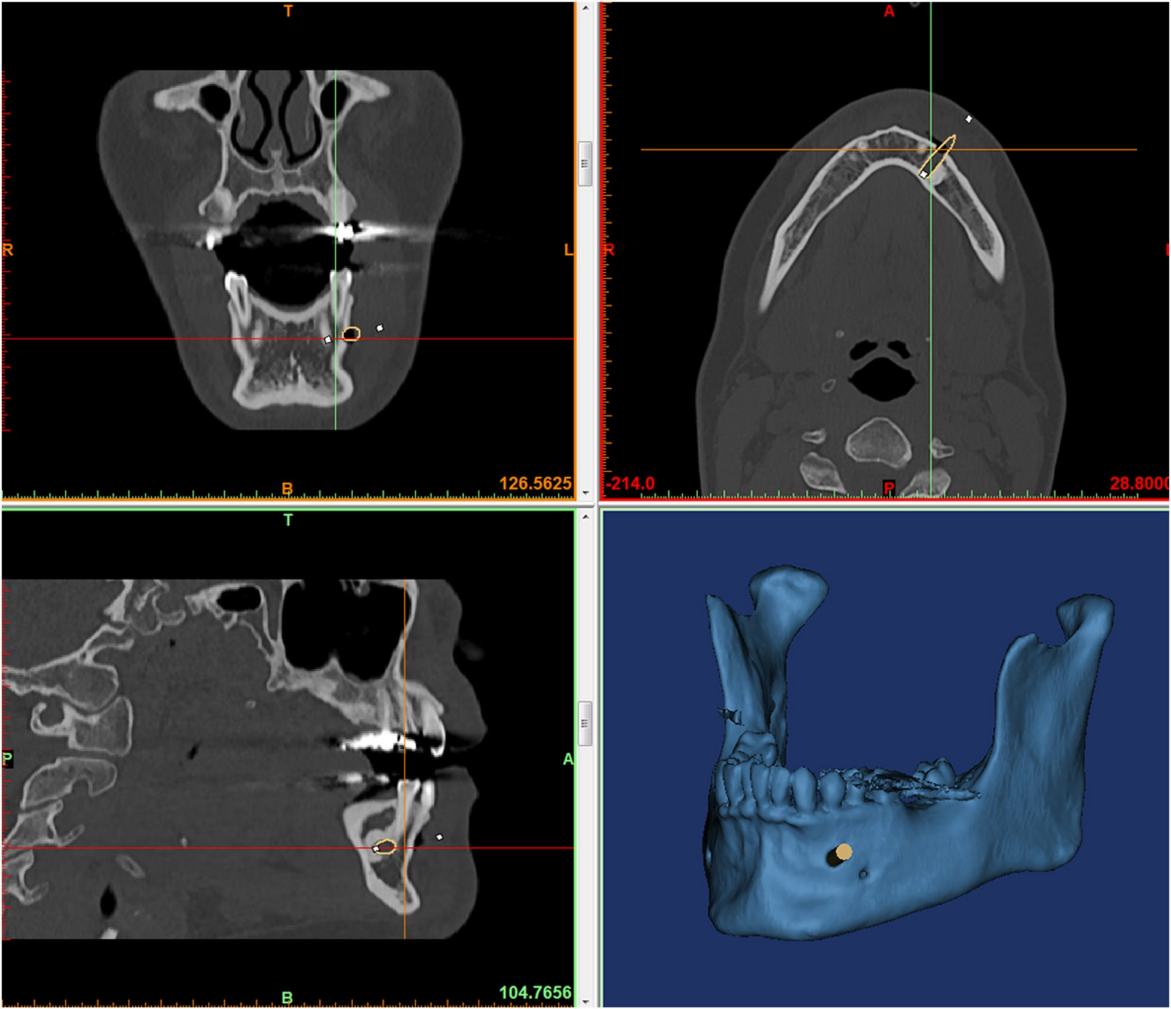
Screenshot of planning the biopsy channel according to CT data

Preoperative CT scans of the patient’s jaws were performed on a Siemens Somatom Force CT scanner (120 kV, 330 mAs, collimation 64 × 0.6 mm, pitch 0.55, slice 0.75 mm) for the osseous and dental situation in relation to the lesion. An impression with alginate was taken to fabricate a plaster cast. After scanning the cast with a 3D-scanner (D750, 3shape, Copenhagen, Denmark), the surface information of the lower jaw including the soft tissues was available in a STL (Standard Tessellation Language) data format. In a 3D-medical image processing software for image segmentation and creation of 3D-models (Mimics Innovation Suite, Materialise, Leuven, Belgium), the DICOM (Digital Imaging and Communications in Medicine) data of the CT scan were segmented, providing a STL data format for the osseous and dental information. The biopsy channel was then designed with an adequate distance to all neighboring anatomical structures such as nerves and tooth roots (Fig. 1). This was followed by a fusion of this data (originating from the CT scan) with the surface data (originating from the alginate impression) using the dental surface information.

**Fig. 2:**
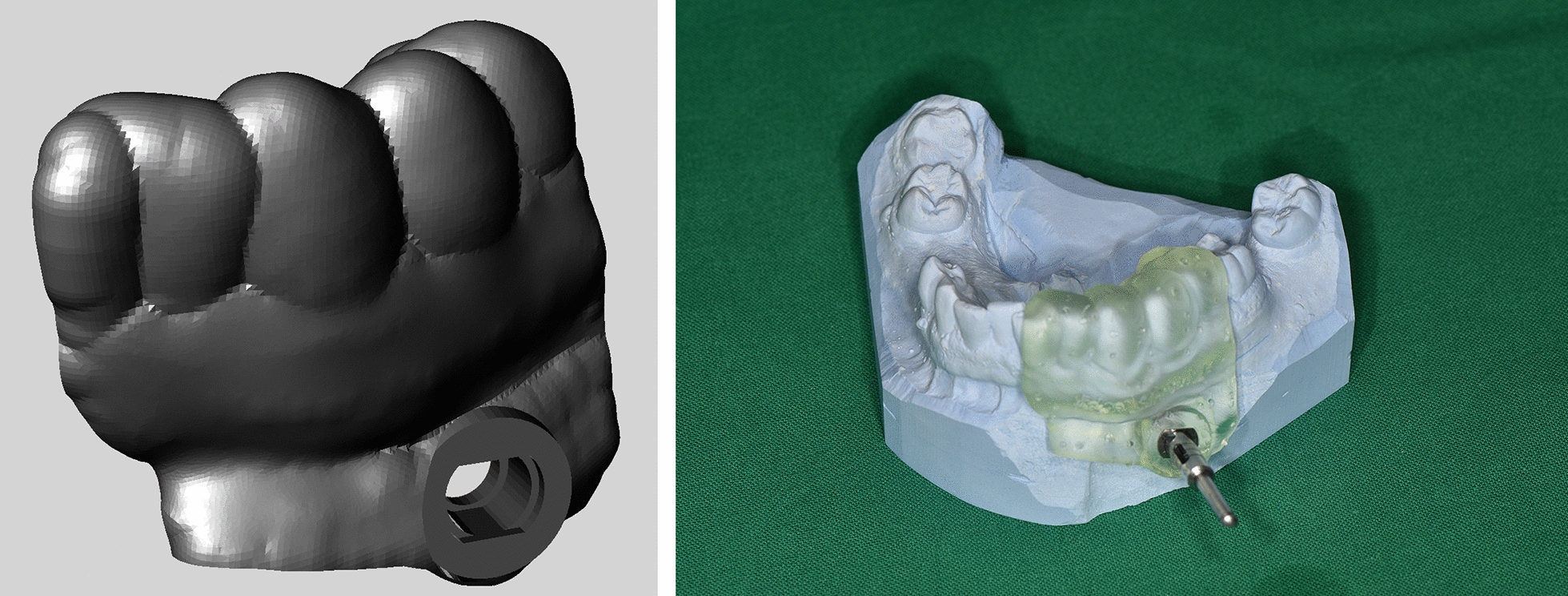
Screenshot of the template after designing in the CAD program is shown in the image on the left and a plaster cast with a fitted surgical guide can be seen in the image on the right. The trephine drill is also shown

The surgical template was then designed (Fig. 2). The depth of the biopsy channel was determined via a limit stop: the head of the contra angle (NSK S-Max SG20, NSK-Nakanishi, Tochigi, Japan) stopped at the surface of the surgical template.

### 3D-printing of surgical templates

After implementation of the STL Data in the printer software (PreForm Software, Formlabs Inc., Somerville, Massachusetts, USA), the surgical template was produced by a 3D-printer (Form 2, Formlabs Inc., Somerville, Massachusetts, USA) using stereolithography with a biocompatible class 1 resin. A metal sleeve was inserted into the templates to avoid plastic particle contamination during biopsy. The accuracy of fit of the templates was verified on the respective plaster models (Fig. 2).

### Biopsy

**Fig. 3:**
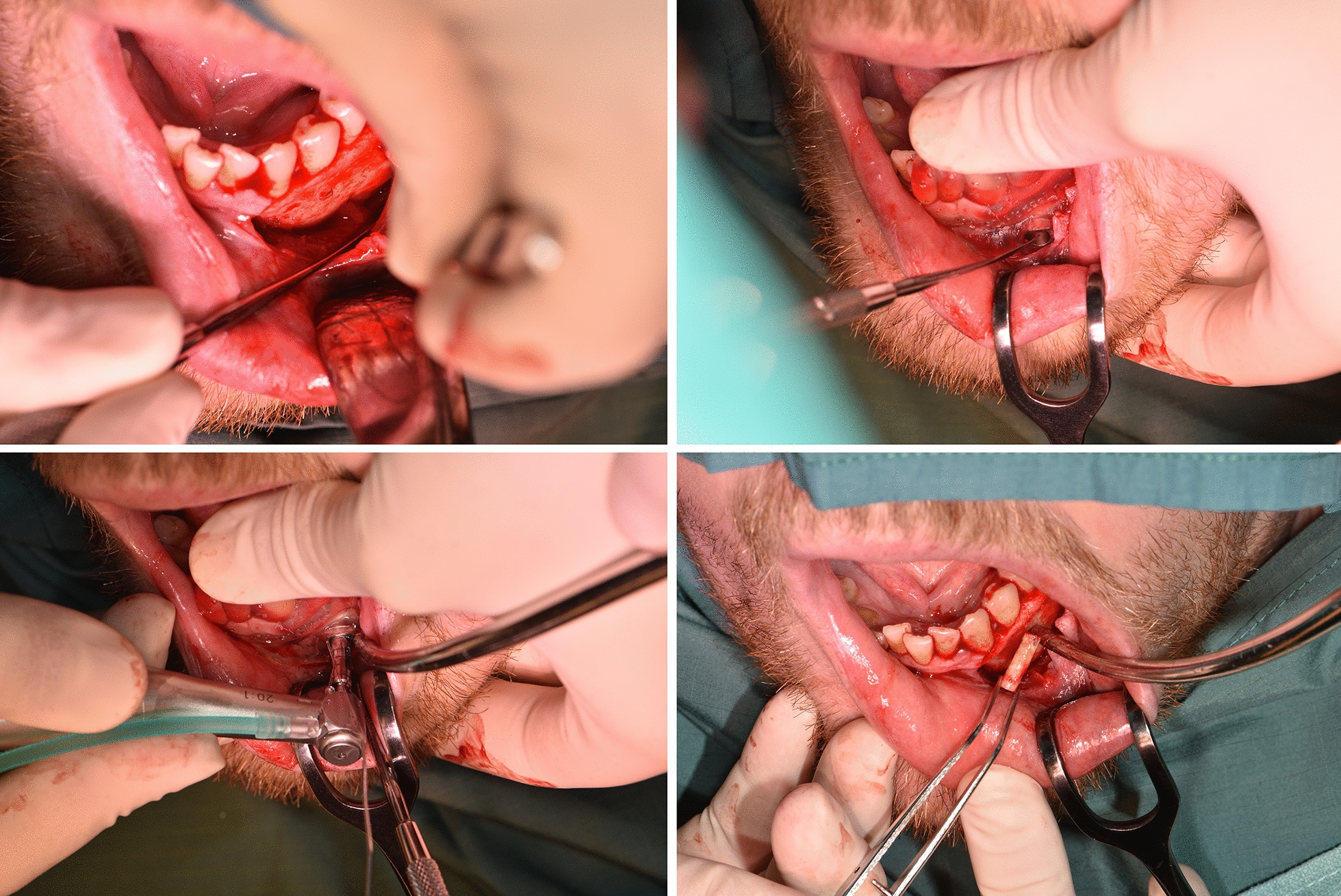
The figure shows certain steps of the biopsy (patient group)

After the patient received local anesthesia, the 3D-printed surgical template was attached to the lower jaw and the entry point of the biopsy channel was marked on the mucosa. The template was removed for performing a suitable approach. Using a trephine drill (XiVE Trephine Drill, inner diameter 3.0 mm, outer diameter 4.2 mm, Dentsply, York, Pennsylvania, USA) and the surgical template, the biopsy was taken (Fig. 3). The bone tissue was sent to the department of oncology for histopathological evaluation.

### Postoperative CT scan

**Fig. 4:**
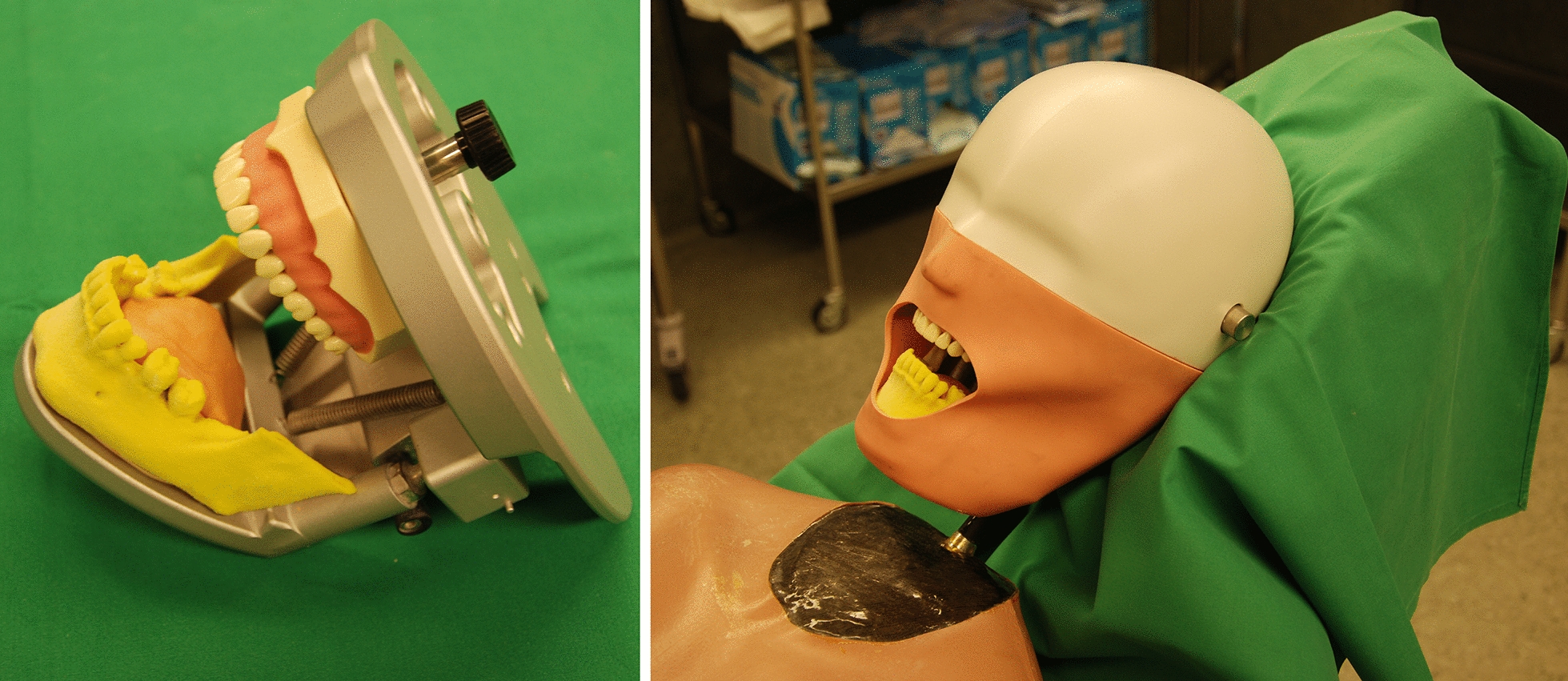
Customized lower jaw models were mounted in a phantom head. The lower part of the phantom head is shown in the image on the upper left. A mask representing facial soft tissues was added to the phantom head which was mounted on a phantom torso. The entire construction was placed on an operating table and covered with surgical drapes, simulating the usual operating room conditions

A postoperative CT scan was performed under the same conditions as the preoperative scan and on the same CT scanner to verify that a representative sample of the lesion could be acquired (Fig. 4).

### Evaluation

**Fig. 5:**
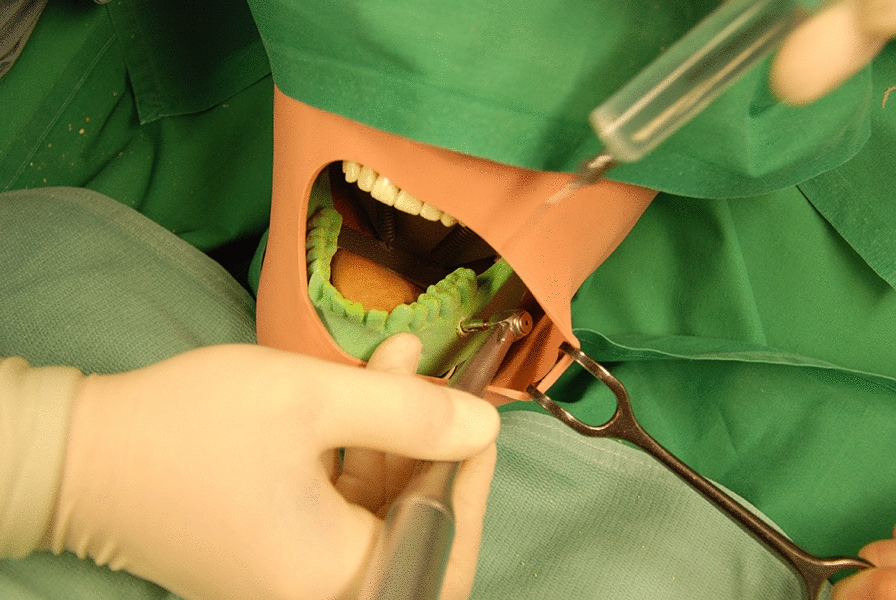
The figure shows certain steps of the biopsy of a customized 3D-printed models of the lower jaw (control group)

The data of the postoperative CT scans were segmented and loaded into Mimics Innovation Suite (Materialise, Leuven, Belgium) (Fig. 5). A fusion of these postoperative data with the preoperative planning data was performed. For determining the axis deviation, the maximum distance between the planned biopsy channel and the true biopsy channel was measured according to the ISO 1101 standard [24], a method which has also been applied in previous studies [5, 25, 26]. Further parameters were determined, such as the angle between the planned and the true biopsy axis as well as the depth of the drilled biopsy channels which was compared to the planned depth.

### Control group

**Fig. 6:**
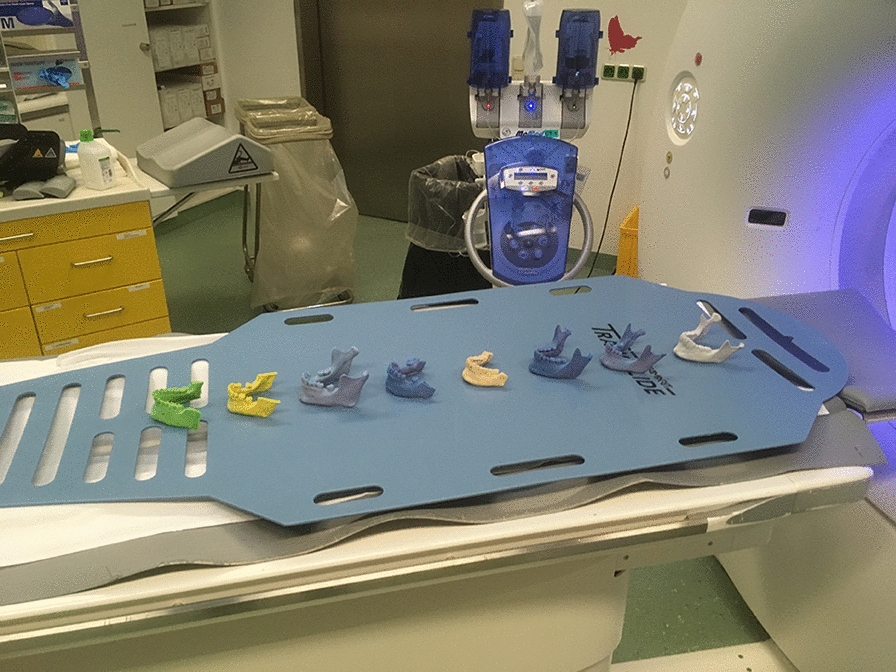
The figure shows customized 3D-printed models in the CT scanner

Customized bone models of the patients’ lower jaws were produced by a 3D-printer (ZPrinter 650, 3D Systems, Rock Hill, South Carolina, USA) according to the CAD data (STL) of the preoperative CT scans. Using these lower jaws with identical dental and bone geometries, free-handed biopsies were performed 1 month after the guided biopsies according to the preoperative CT data and planning. All procedures in this study were performed by the same experienced surgeon. The models were mounted in a phantom head (Frasaco P6 phantom head, frasaco GmbH, Tettnang, Germany) to simulate operation room conditions and the phantom head was mounted on a torso and placed on the operating table (Fig. 4). The operating area was then covered in the usual way to simulate a hands-free surgery in the operating room as realistically as possible. An identical equipment was used as for the guided biopsies (Fig. 5). CT scans of the customized models were performed using the same parameters as for the previous CT scans (Fig. 6). The deviation of the free-handed biopsies was evaluated in the same manner as described for the procedures using surgical templates.

### Statistics

The software IBM SPSS Statistics 22 (IBM, Armonk, New York, USA) was used to calculate mean values (arithmetic mean) and standard deviations. Data were tested for normal distribution using the software Sigma Stat 3.1 software (Systat Inc, Chicago, IL, USA). A Welch’s *t*-test (two-sample unpooled *t*-test for unequal variances) was performed to compare the biopsies of the test group and the control group using the QuickCalcs software (GraphPad Software, Inc. La Jolla, California, USA).

## Results

The clinical outcomes of the 8 included patients are shown in Table 1. The lesions were classified according to the 4th Edition of the World Health Organization Classification of Head and Neck Tumours [27]. One patient developed a submucosal abscess, which healed without permanent damage after vestibular incision and temporary iodine strip insertion.

**Table 1:** Patient data: overview of the patients’ data on the location of the biopsy, diagnosis after pathological examination and complications after the surgery

Patient	Location	Diagnosis	Complication
1	46/47	Simple bone cyst	
2	46/47	Odontoma	
3	Supramental	Sclerosis zone	
4	37	Sclerosis zone	
5	47	Odontoma	Submucosal abscess
6	34	Sclerosis zone	
7	46	Fibrous dysplasia	
8	36/37	Odontoma	

**Fig. 7:**
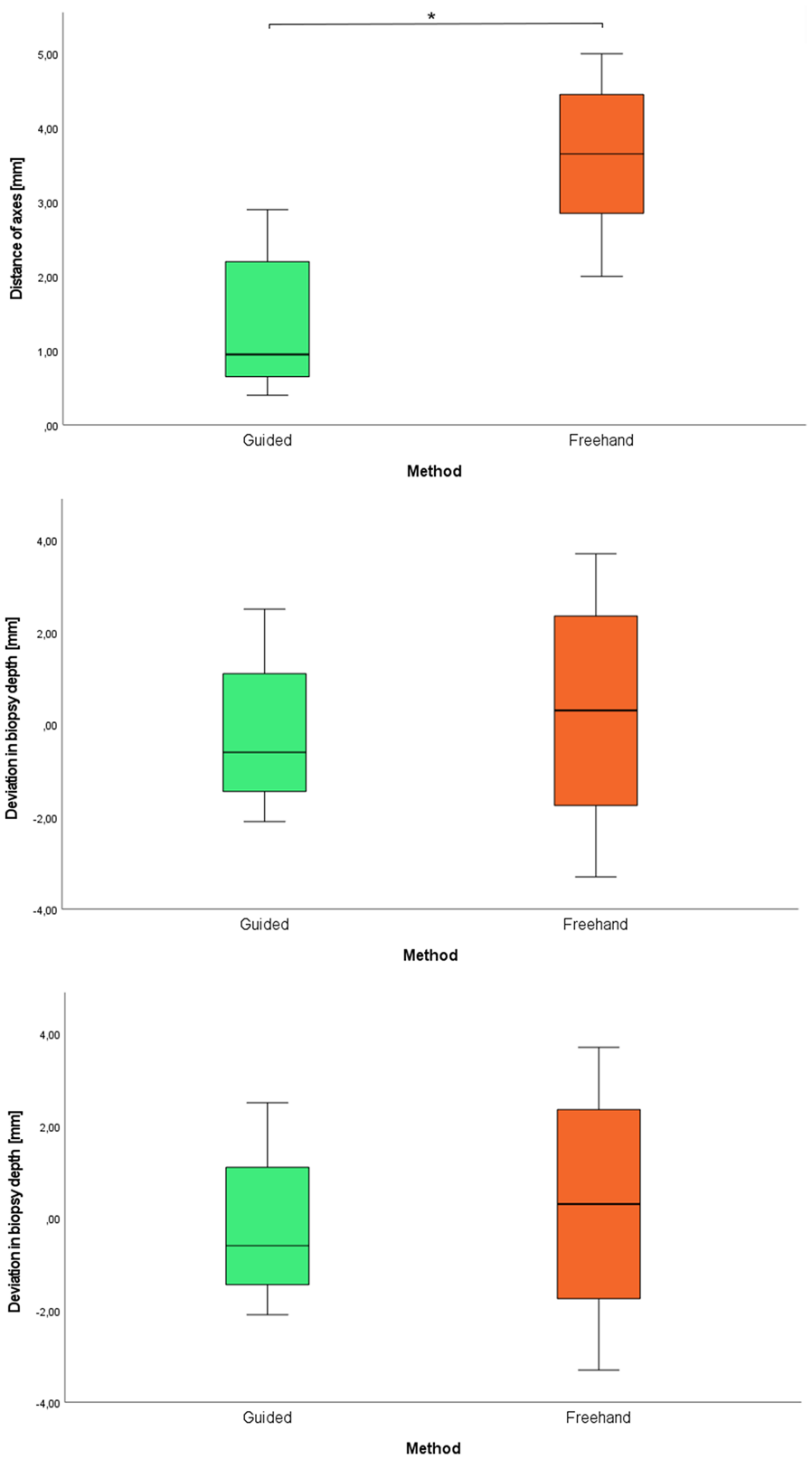
Box plot showing the maximum axis deviation for the guided and the free-handed group. Data presented as medians and interquartile ranges (IQR) with whiskers extending to a maximum of 1.5 × IQR. (*P = 0.0005)

The mean deviation of the biopsy axes was 1.4 ± 0.9 mm for the biopsies with a surgical guide and 3.6 ± 1.0 for the free-handed biopsies (Fig. 7). The data showed a normal distribution and statistical comparison of the groups revealed a significant difference (*p*  = 0.0005).

The average biopsy angle deviation was 6.8 ± 4.0 for template biopsies and 15.4 ± 3.6 for free-handed biopsies (Fig. 7). The difference of the normally distributed data was again statistically significant (*p*  = 0.0005).

The difference between the planned depth of biopsy channels and the true depth of biopsy channels was—0.2 ± 1.6 mm for biopsies with surgical guides and 0.3 ± 2.5 mm for free-handed biopsies (Fig. 7). These data showed a normal distribution again, but no significant difference was found (*p*  = 0.6668). Data are summarized in Table 2.

**Table 2:** Deviation of biopsy channels

	Guided biopsies	Free-handed biopsies
Maximum distance of axes	1.4 ± 0.9 mm	3.6 ± 1.0 mm
Angle deviation	6.8 ± 4.0	15.4 ± 3.6
Depth deviation	− 0.2 ± 1.6 mm	0.3 ± 2.5 mm

## Discussion

This study examined if 3D-printed surgical guides are a suitable tool for osseous biopsies of the lower jaw in vivo. Free-handed biopsies performed on customized 3D-printed models of the lower jaws served as control groups for evaluating the accuracy of the guided biopsies.

The guided group showed more accurate results than the free-handed group for the biopsy axis as well as for the biopsy angle. No significant difference between the two groups was found for the accuracy of the biopsy depth. Compared to the angular deviation (4.35 ± 2.5) and depth deviation (− 1.40 ± 1.4 mm) of the recently published study from Lotz et al. [20], the present study found a slightly higher angular deviation (6.8 ± 4.0) and a slightly smaller depth deviation (− 0.2 ± 1.6 mm). Unfortunately, Lotz et al. did not include an evaluation of the deviation of the biopsy axes and there was no free-handed control group [20]. However, the deviation of the biopsy axes (distance of the planned biopsy axis from the true biopsy axis) is an important parameter, since a large axis deviation can cause a false biopsy (failing to hit the tumour), even if the angular deviation and the depth deviation are small. Since no other studies considered this parameter, the results of this present study were compared to data from studies examining the accuracy of dental implant positioning with 3D-printed surgical guides. A mean axis deviation of < 1 mm and a mean angular deviation of < 3 have been reported for dental implants, however, the distance between the planned and true implant position was measured at the abutment level of the implants, which is the outermost point of the implant after insertion [28]. This is a plausible method for dental implants, but not for biopsies, as the relevant deviation measurement is at the end of the drilled biopsy channel. Nevertheless, the apical deviation measured in the present study is within the range of results reported in previous implant studies (Cassetta et al., Van de Wiele et al. [13, 21]). On the other hand, the measured angles in the present study are larger compared to those in the implant studies. A possible explanation for this observation may be the greater distance between the drill channel and the teeth by which the surgical templates are supported during the procedure. This consideration is well understood when looking at studies with similarly manufactured surgical templates for dental implant placement [18]. Furthermore, the use of surgical templates did not significantly improve the accuracy of the biopsy channel depth, which indicates that reading the scale on the trephine drills is a reliable method for determining a precise channel depth. Nonetheless, the guided biopsies tended to be less deep and there was a lower standard deviation. In a larger cohort of patients, there might be a significant difference in terms of depth. Based on the study results (− 0.2 ± 1.6 mm), it is recommended to consider the potential deviation in depth and thus plan the bone biopsies a little deeper to capture the lesions safely, if the individual anatomy allows it.

Using 3D-printed lower jaws as controls instead of a patient control group presents a limitation of this study, but it is a common method for generating a control group in computer-aided surgery [26]. Naturally, it was easier to access the dental and bone geometries in the control group as these structures were not covered by any soft tissue. In addition, the jaws were freely movable and could be positioned as needed. Altogether, the free-handed biopsies in the control group were performed under ideal conditions. This makes a representative comparison of the operation time between the groups difficult. The ideal control group for this study would have been a clinical control group with patients. If such a clinical control group had been chosen, the free-handed biopsies would have been more demanding and thus presumably less accurate under clinical conditions. This would lead to larger significant differences between the guided group and the clinical control group.

In contrast, the present control group also has some advantages. The dental and osseous geometry of the 3D-printed lower jaws was identical to the lower jaws of the study group. In addition, the lesions in the control group were assumed to be at the same locations as in the study group, allowing the clinicians to use the same planning for the study group and the control group. This reduces the potential bias due to different locations and directions of the biopsies as well as different geometries of the jaws. The errors of the single steps (e.g., scanning, fusion, printing the guides) were not evaluated. However, this study should evaluate the final result. The number of biopsies was limited and therefore larger randomized studies should be planned in the future.

## Conclusions

In conclusion, the null hypothesis (H0) was rejected for the distance of axes and the angle deviation and this study showed that surgical guides that were produced with a stereolithographic desktop 3D-printer allow significantly higher accuracy of biopsies.

## Data Availability

Not applicable.
